# The Tetramethylpyrazine Derivative Statmp-151: A Novel Small Molecule Stat3 Inhibitor With Promising Activity Against Breast Cancer

**DOI:** 10.3389/fphar.2021.651976

**Published:** 2021-04-15

**Authors:** Chen Fan, Yijie Wang, Hui Huang, Wenzhen Li, Jialin Ma, Dongping Yao, Zijun Tang, Taixiong Xue, Liyang Ha, Yan Ren, Yiwen Zhang, Qin Wang, Yongmei Xie, Yi Luo, Rui Tan, Jian Gu

**Affiliations:** ^1^ College of Pharmacy, Southwest Minzu University, Chengdu, China; ^2^ State Key Laboratory of Biotherapy and Cancer Center, Department of Orthopedics, West China Hospital, Sichuan University, Chengdu, China; ^3^ Department of Oncology, The Fifth Hospital of Wuhan, Wuhan, China; ^4^ College of Life Science and Engineering, Southwest Jiaotong University, Chengdu, China

**Keywords:** breast cancer, tetramethylpyrazine derivatives, stat3, statmp-151, apoptosis

## Abstract

Breast cancer is the most common malignancy in women and is a molecularly heterogeneous disease. Signal transducer and activator of transcription 3 (Stat3) is overexpressed and hyperactivated in a variety of human tumours, including breast cancer, thus representing a promising target for breast cancer treatment. In the present study, we evaluated the activities of a novel Stat3 inhibitor named Statmp-151 in the human breast cancer cell lines MCF-7 and MDA-MB-231 and the murine mammary carcinoma cell line 4T1. The *in vitro* results showed that Statmp-151 inhibited the proliferation of breast cancer cell lines in a dose- and time-dependent manner and suppressed the phosphorylation of Stat3 in a dose-dependent manner. Flow cytometry (FCM) assays revealed that Statmp-151 affected mitochondrial membrane potential and reactive oxygen species (ROS) production. Furthermore, Statmp-151 inhibited cell migration, as shown by analysis of the matrix metalloproteinases MMP2 and MMP9. Finally, in a 4T1 tumour-bearing mouse model, intraperitoneal injection of 30 mg/kg/day Statmp-151 significantly suppressed the growth of tumours without obvious toxicity. These results indicated that Statmp-151 might be a potential candidate for the treatment of breast cancer.

## Introduction

Breast cancer is a major cause of cancer-related mortality as one of the most common cancers in females. The strong metastatic potential of this disease accounts for most deaths from breast cancer. Treatment for breast cancer is multidisciplinary, including locoregional surgery and radiotherapy as well as systemic treatment (Harbeck et al., 2019). Triple-negative breast cancer (TNBC) is the most aggressive type and has much higher recurrence and metastasis rates than other types (Waks and Winer, 2019). When patients are diagnosed with TNBC at the early stage, a combination of chemotherapeutic agents with radiotherapy or no radiotherapy is used as the standard nonsurgical treatment; unfortunately, the efficacy is limited (Sharma and Priyanka, 2018). In addition to chemotherapy and surgery, molecular-targeted therapy has been one of the hotspots in the field of breast cancer research in recent years (Lu and Liu, 2020).

Stat3 regulates tyrosinase gene expression and transcript activity and thus plays a pivotal role in promoting breast cancer growth and metastasis (Federica et al., 2018). The Stat3 protein consists of an *N*-terminal domain, a coiled-coil domain (CCD), a DNA binding domain (DBD), a linker domain, an SRC homology 2 (SH2) domain for phosphorylation and dimerization, and a C-terminal transactivation domain (TAD) (Furtek et al., 2016). The SH2 domain is a very important domain of Stat3 and is related to many important physiological functions of Stat3. When extracellular signals are transmitted to the cell membrane, through a series of cascade amplification reactions, tyrosine 705 (Tyr705) of the SH2 domain is finally phosphorylated to activate the entire Stat3. The activated Stat3 monomer dimerizes through the SH2 domain and enters the nucleus, binding with DNA and promoting the expression of downstream genes (Schust et al., 2006; Chen et al., 2013).

Therefore, targeted inhibitors of the SH2 domain may have potential to treat breast cancer. However, targeted inhibitors of the SH2 domain possess many disadvantages, such as high toxicity, low activity and poor selectivity. Tetramethylpyrazine (TMP) is an alkaloid isolated from the rhizome of *Ligusticum chuanxiong* Hort. TMP has been reported to have several significant biological effects, such as antioxidative, antifibrotic, calcium antagonist and antitumour effects (Zhao et al., 2016). TMP could also inhibit the viability of MDA-MB-231 breast cancer cells (Shen et al., 2018). However, the anticancer activity is relatively weak. Hence, it is necessary to enhance its effects *via* reasonable structural modification. In this study, we synthesized Statmp-151 to improve TMP’s anti-breast cancer activity; this molecule combined the classic SH2 domain targeting inhibitor Stattic with TMP (Zeng et al., 2013; Furtek et al., 2016). Finally, the potential anti-breast cancer mechanisms were explored by a series of experiments.

## Materials and Methods

### Cell Culture

The breast cancer cell lines MCF-7, MDA-MB-231 and 4T1 were purchased from the American Type Culture Collection (Rockville, MD, United States). Cells were maintained in DMEM or RPMI 1640 medium supplemented with 10% foetal bovine serum (Si Ji Qin Bioengineering, China) and 1% antibiotics (penicillin and streptomycin) at 37°C in a 5% CO_2_ incubator.

### Cell Viability and Colony Formation Assays

The cells (1-5 × 10^3^ cells per well) were incubated in 96-well plates overnight and then treated with different concentrations of Statmp-151 for 24, 48, and 72 h. Afterward, 5 mg/ml MTT (20 μl) was added to each well for 3 h. Finally, the supernatant was removed, and 150 µl of DMSO was added to each well. The optical density values were determined at 490 or 570 nm by a SpectraMAX M5 Microplate Spectrophotometer. All experiments were performed three times with three replicates.

Colony formation was measured by seeding cells in 6-well plates at 500–800 cells per well and treating the cells with various concentrations of Statmp-151 after approximately 24 h of incubation. The culture medium containing Statmp-151 was replaced every three days. All cells were fixed and stained with 0.5% crystal violet after 12 days.

### Apoptosis Analysis

For determination of the effect of Statmp-151 on tumour apoptosis, apoptosis detection kits were used. The cells (1 × 10^5^ cells per well) were incubated in 6-well plates overnight and then treated with different concentrations of Statmp-151. After 24 h, the cells were harvested and washed twice with cold PBS. Following the manufacturer’s instructions, the cells were stained with FITC-conjugated Annexin V and PI (Propidium Iodide) and detected by FCM.

### Mitochondrial Membrane Potential (Δψm)

The cells (1 × 10^5^ cells per well) were incubated in 6-well plates overnight, and treated with Statmp-151 for another 24 h. Then, the cells were harvested and washed twice with cold PBS, stained with JC-1 according to the instructions, and finally detected by FCM.

### Reactive Oxygen Species Level in Cells

The cells (1 × 10^5^ cells per well) were incubated in 6-well plates overnight and then treated with different concentrations of Statmp-151. After 24 h, the cells were harvested and washed twice with cold PBS. The ROS levels were monitored using 10 µM DCFH-DA for 30 min and then detected by FCM.

### Western Blotting

For analysis of the expression of the corresponding proteins in MDA-MB-231 and 4T1 cells after incubation with Statmp-151 for 24 h, harvested cells were lysed with RIPA buffer for 1 h. Then, the protein concentrations were measured and equalized. SDS-PAGE with the optimal concentration of proteins selected according to their molecular weight was performed, and then, the proteins were transferred onto polyvinylidene difluoride nitrocellulose membranes. After incubation with 5% skim milk for 1 h, the target protein was incubated with the corresponding primary antibodies overnight at 4°C. The next day, the cells were washed several times and then incubated with the corresponding secondary antibodies, followed by washing several times. Finally, the protein bands were visualized using an enhanced chemiluminescence kit. Monoclonal β-actin proteins were used as a reference.

### Wound-Healing Migration Assays

A wound-healing migration assay was performed to evaluate cell migration. When cells grew to 80% confluence in 6-well plates, they were renewed with 2% FBS containing different concentrations of Statmp-151. Images were taken at 0 and 24 h by an inverted microscope.

### The Anticancer Effect of Statmp-151 *In Vivo*

All animal experiments were conducted in accordance with the principles and procedures approved by the Committee on the Ethics of Animal Experiments of State Key Laboratory of Biotherapy, Sichuan University. BALB/c mice aged 6–8 weeks were purchased from the experimental Animal Centre of Sichuan University. Approximately 1 × 10^6^ 4T1 cells were inoculated into the right lower limb of the mice. We randomly divided the mice into 4 groups (*n* = 5) when the tumours reached an average volume of 100 mm^3^. Statmp-151 (10, 20, and 30 mg/kg) or vehicle was intraperitoneally injected once a day for 15 days. After administration, the body weights and tumour sizes of the mice were measured every 3 days. At the end of the animal experiment, the weight of the tissue and tumour was recorded, and then, the tumour volume was measured and photographed. The tumour tissue was stored in paraformaldehyde for further analysis.

### Toxicity Evaluation

For evaluation of the safety of Statmp-151 during the treatment, all the mice were observed continuously for general conditions, such as body weight, appetite, mental state and other clinical features. Blood was obtained from the eyeball for biochemistry analysis. Haematoxylin and eosin staining was performed of paraffin sections of lung, liver, spleen, heart and kidney tissues of the mice treated with Statmp-151.

### Statistical Analysis

The results are presented as the mean and standard deviation (M±SD). Statistically significant *p* values were labelled as follows: *p < 0.05; **p < 0.01; ***p < 0.001. All statistical workflows were performed in GraphPad Prism (version 7.0).

## Results

### Drug Design of Statmp-151

**FIGURE 1:**
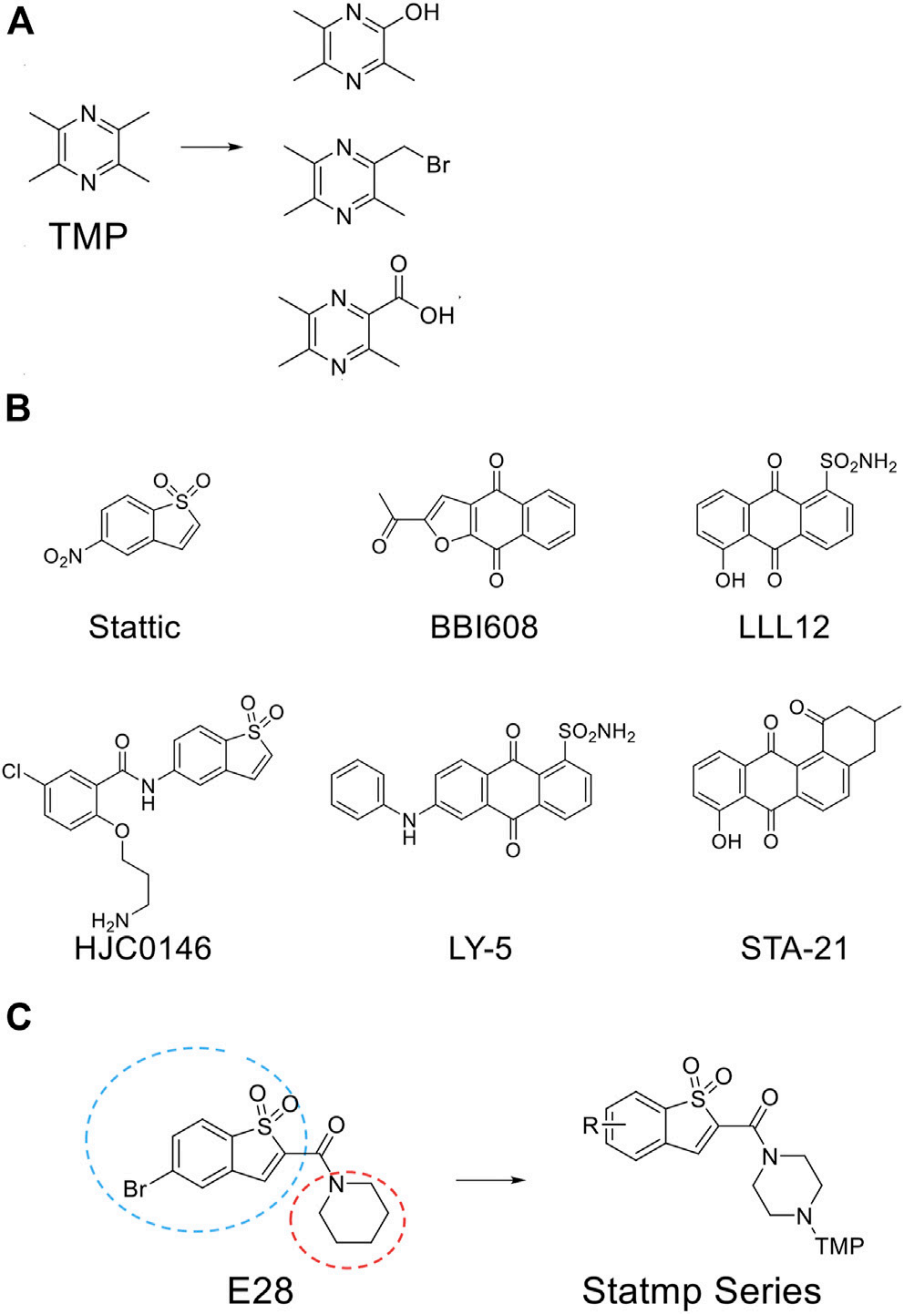
The chemical structure involved in this study. **(A)** Three common fragments of TMP derivatives. **(B)** Some current reported Stat3 inhibitors. **(C)** From E28 to Statmp series which was *via* rational drug design and virtual screening.

**FIGURE 2:**
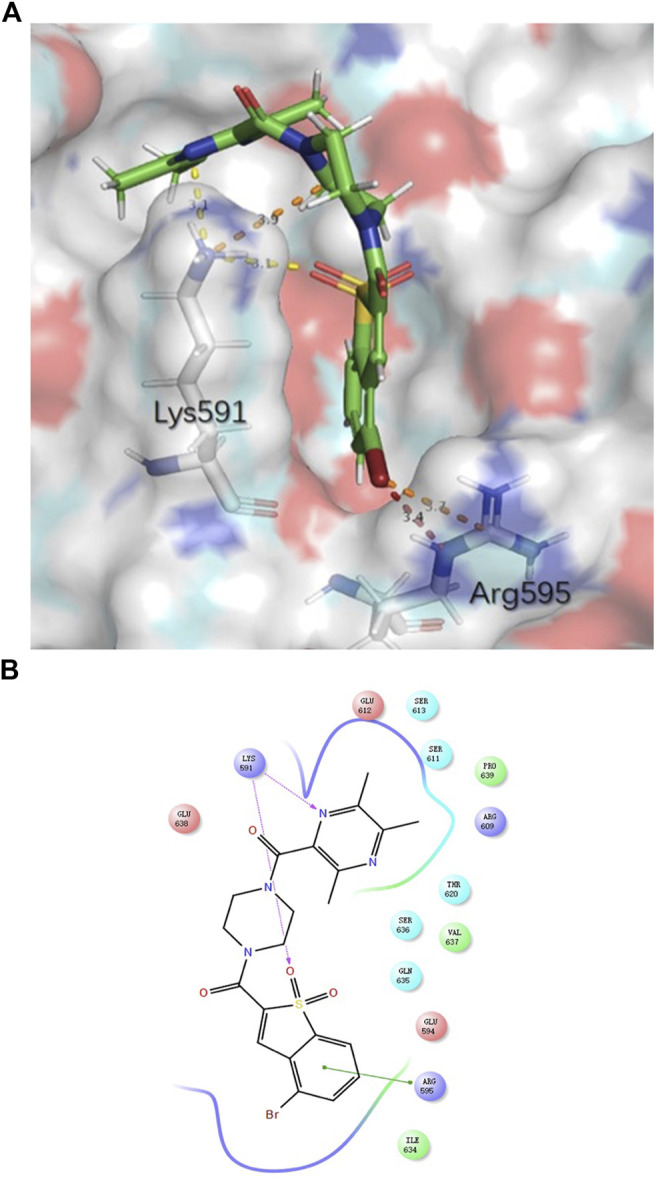
The binding mode of Statmp-151 with Stat3 pocket. **(A)** Statmp-151 possesses a favourable pose interacting with Stat3 residues. **(B)** The interaction diagram for Statmp-151 interacting with Stat3 residues.

TMP was reported to possess multiple activities, as described above. For chemical structure modification, there are three common fragments of TMP to utilize (Figure 1A). Carboxyl, bromo and hydroxyl groups are operable reactive groups for various modification strategies. Stat3-targeted inhibitors, such as Stattic, BBI608, LLL12, HJC0146, LY-5 and STA-21, are listed in Figure 1B. Among them, Stattic was the first small molecule inhibitor of Stat3 activation and dimerization (Schust et al., 2006). This compound is often used for further development to discover Stat3 inhibitors (Ji et al., 2015). In this study, we used (5-bromo-1,1-dioxidobenzo[b]thiophen-2-yl) (piperidin-1-yl) methanone (E28) (Ji et al., 2015) as the lead compound to discover Stat3 inhibitors containing TMP. We replaced the piperazine ring of E28 with a piperazine ring for further modification (in blue circle) and explored the group linking TMP with the piperazine ring. In terms of the scaffold of Stattic, we also explored the substituents on the benzene ring (in red circle), and a total of 38 compounds were designed (Figure 1C). We employed the Glide docking program to conduct a virtual screening against these 38 rationally designed Statmp series and finally found that Statmp-151 could exhibit potent activity against Stat3, had the best docking score and showed a more favourable conformation (Figure 2). Binding mode analysis indicated that the pyrazine ring and sulfone form hydrogen bonds with Lys591, while the bromo substituent forms halogen bonds with Arg595. Then, wet laboratory experiments were conducted to explore whether Statmp-151 was consistent with the primary drug design. The synthesis and characterization of Statmp-151 are shown in the supporting information.

### Statmp-151 Inhibited the Growth of Breast Cancer Cells *In Vitro*

**FIGURE 3:**
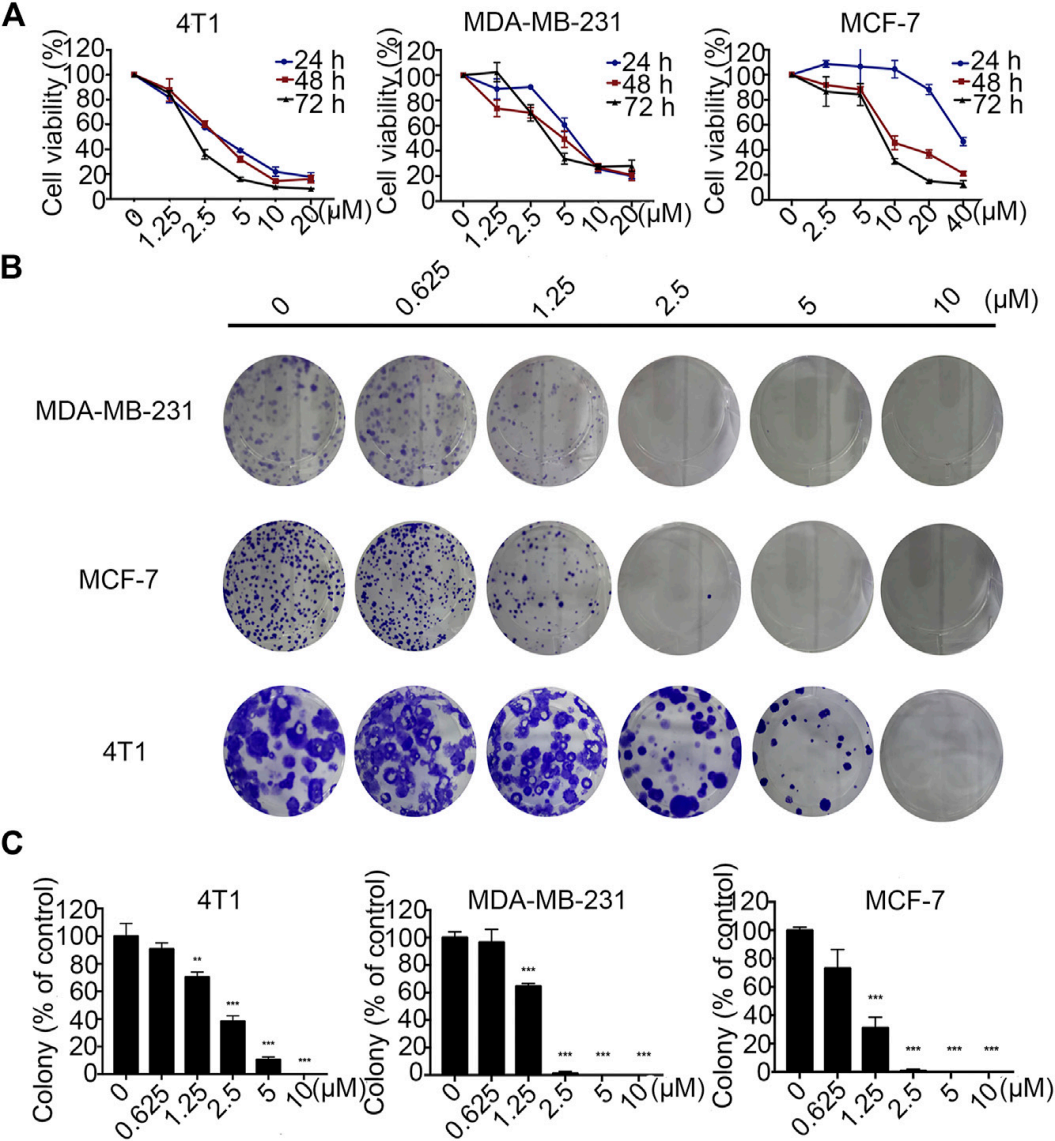
Statmp-151 inhibited the growth of breast cancer cells *in vitro.* **(A)** Breast cancer cell lines 4T1, MDA-MB-231 and MCF-7 were treated with different concentrations of Statmp-151 for 24, 48 and 72 h, respectively. **(B)** The effects of statmp-151 on the colony formation in three breast cancer cell lines for 10 days. **(C)** The statistical results were presented using vehicle control at 100%. Each point represents the mean ± SD for at least 3 independent experiments (*p < 0.05, **p < 0.01 and ***p < 0.001 vs. control group).

The effects of Statmp-151 on the proliferation of MCF-7, MDA-MB-231 and 4T1 cells were detected by MTT assays (Liu et al., 2019). In 4T1 and MDA-MB-231 cells where Statmp-151 was used at concentrations of 0–20 µM, and in MCF-7 cells where Statmp-151 was used at concentrations of 0–40 µM for 24, 48 and 72 h. The results showed that the IC_50_ at 72 h was 2.20 ± 0.01 µM in 4T1 cells, 4.75 ± 0.39 µM in MDA-MB-231 cells, and 8.06 ± 0.08 µM in MCF-7 cells (Figure 3A). Pan J reported the anti-tumour activity of TMP which is not ideal. The ligustrazine IC_50_ results showed it is about 10 mmol/l in breast cancer. The above results significantly improved the anti-tumour activity of maternal TMP (Pan et al., 2015).

A clone formation experiment was utilized to further verify the effects of Statmp-151 on the proliferation of MDA-MB-231, MCF-7 and 4T1 cells. As shown in Figure 3B,C, cancer cell proliferation was reduced in a concentration-dependent manner, which was consistent with the results of the MTT assay.

### Statmp-151 Induced Apoptosis of Breast Cancer Cells

**FIGURE 4:**
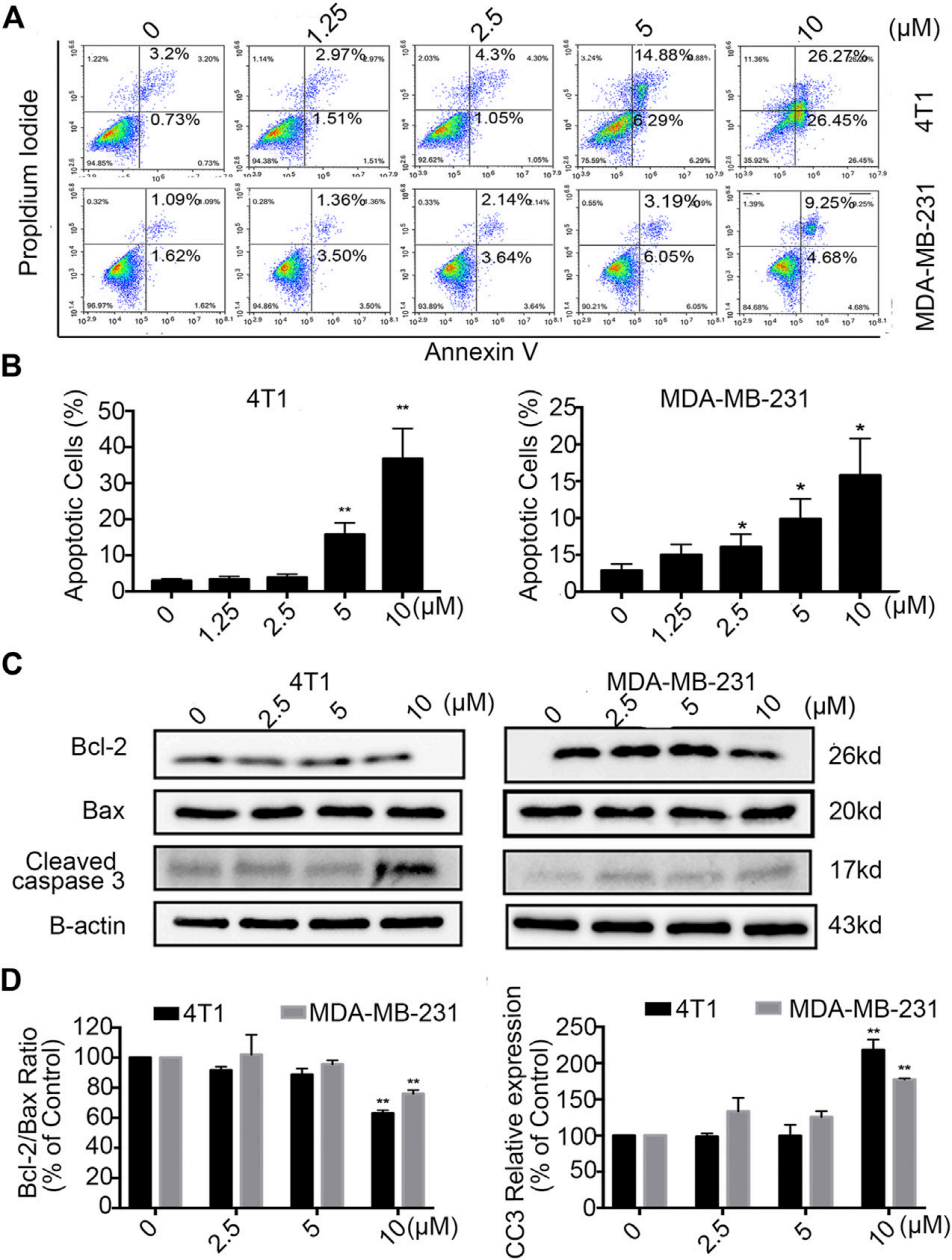
Statmp-151 induced apoptosis in breast cancer cells. **(A)** 4T1 and MDA-MB-231 cells were treated with different concentrations of Statmp-151 for 24 h and then analyzed by FCM using Annexin V/PI dual staining. (B) The apoptosis data analysis is presented in a histogram. **(C)** After treated with Statmp-151 for 24 h in 4T1 and MDA-MB-231 cells, the expressions of cleaved caspase-3, Bcl-2 and Bax were obtained, with β-actin used as a standard control. (**D**) The gray level statistics were quantified with ImageJ (*p < 0.05, **p < 0.01 and ***p < 0.001 vs. the control group).

For further analysis of the effect of Statmp-151, we performed Annexin V and PI double dye detection of MDA-MB-231 and 4T1 cells (Zhu et al., 2016). As shown in Figure 4A,B, Statmp-151 was able to induce breast cancer cell apoptosis in a dose-dependent manner after treatment for 24 h compared to that in the control group.

To confirm the apoptotic effect of Statmp-151 on breast cancer cells, we determined the expression of Bcl-2, Bax and Cleaved caspase-3 (CC3) in MDA-MB-231 and 4T1 cells after treatment with Statmp-151 for 24 h. As shown in Figure 4C,D, the expression level of Bcl-2 was strongly decreased in 4T1 cells, Bax expression was not changed, and CC3 expression was significantly increased. This treatment showed no effect on Bcl-2/Bax ratio expression and increased CC3 expression in MDA-MB-231 cells. Collectively, these results showed that Statmp-151 could induce breast cancer cell apoptosis.

### The Effect of Statmp-151 on Mitochondrial Membrane Potential and ROS

Mitochondrial membrane potential is a marker of the early apoptotic pathway. During mitochondrial pathway-mediated apoptosis, mitochondrial membrane permeability increases, stromal calcium outflow and mitochondrial membrane rupture occur, and apoptotic induction factor (AIF) and cytochrome C are released, thus initiating a cascade reaction to activate the caspase family and ultimately induce apoptosis (Reed, 1997). Mitochondrial membrane potential and intracellular ROS level jointly have a marker effect on the growth and development of cancer cells. When the mitochondrial membrane potential changes or the ROS level changes, it often indicates the loss of homeostasis of cancer cells. Once a compound has an inhibitory effect on cancer cells, these two indicators usually change (Zhao et al., 2016; Burke, 2017).

**FIGURE 5:**
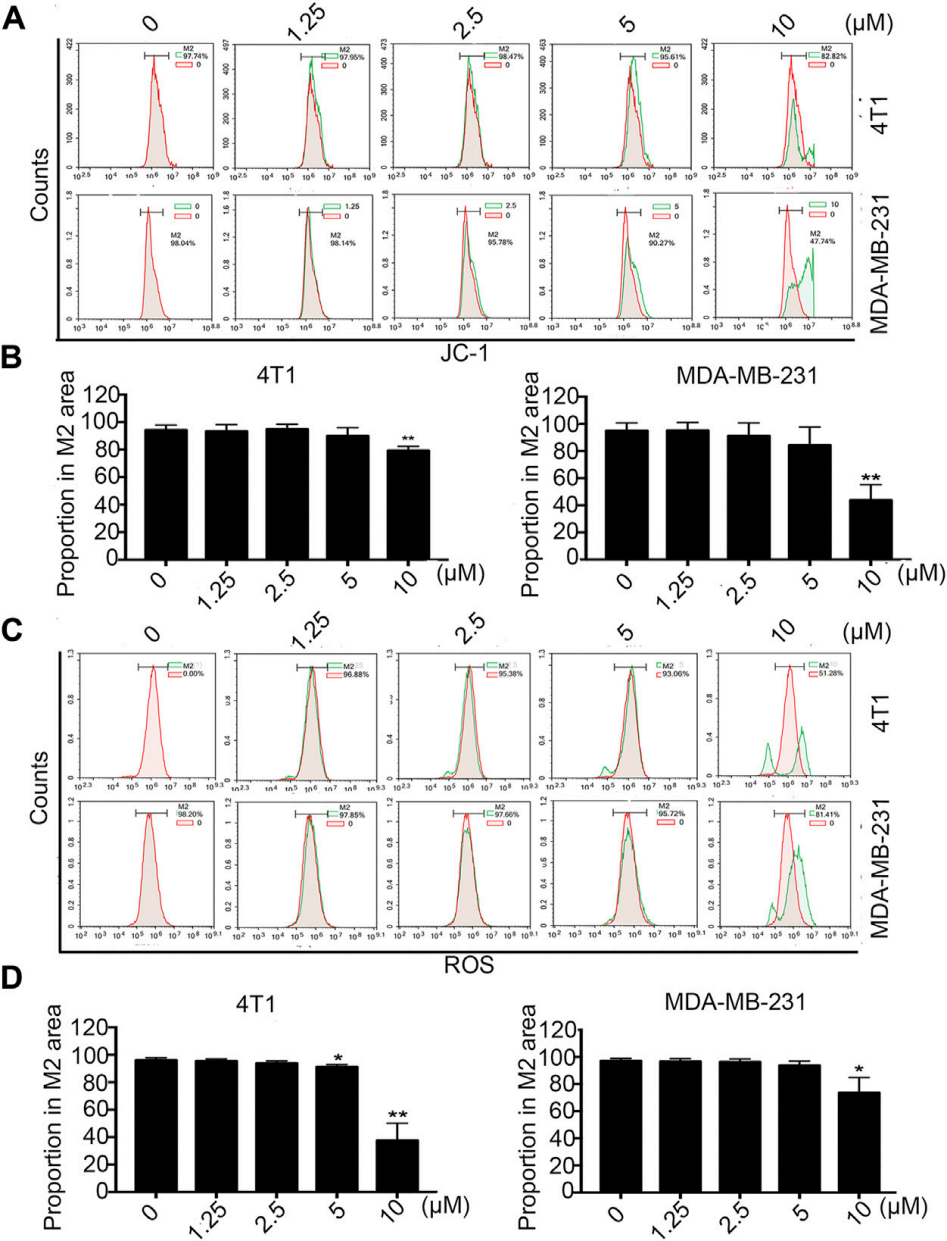
The effect of Statmp-151 on mitochondrial ΔΨm and ROS. **(A)** Statmp-151 decreased the mitochondrial membrane potential in 4T1 and MDA-MB-231 cells. **(B)** The mitochondrial ΔΨm results were presented using quantified. **(C)** The levels of ROS were decreased after treatment with Statmp-151. The harvested 4T1 and MDA-MB-231 cells were measured by FCM. **(D)** The ROS data is presented in a histogram. Data are present as mean ± SD for at least three independent experiments (*p < 0.05, **p < 0.01 and ***p < 0.001 vs. control group).

According to this knowledge, we detected the changes in mitochondrial membrane potential (ΔΨm) by FCM after treatment with different concentrations of Statmp-151 in breast cancer cells. As shown in Figure 5A,B, treatment with 10 µM Statpm-151 led to a 17.28% loss of ΔΨm for 4T1 cells. The loss was 52.26% in MDA-MB-231 cells under the same conditions.

Tumor cells are often in an environment with a high level of reactive oxygen species (ROS), and intracellular ROS levels are closely related to the stability of the mitochondrial membrane potential (Li et al., 2011). The effect of Statmp-151 on membrane potential was identified in previous experiments, and the ligustrazine structure of Statmp-151 has been reported to reduce intracellular reactive oxygen species (Zhang et al., 2010). In view of this, we also determined the effect of Statmp-151 on ROS by FCM using DCFH-DA. The results indicated that Statmp-151 led to increased ROS levels in a dose-dependent manner. As shown in Figure 5C,D, the results showed that after 24 h of treatment with 10 µM Statmp-151 in 4T1 and MDA-MB-231 cells, ROS levels were 18.89 and 48.72%, respectively. These results confirmed that Statmp-151 induced cell apoptosis *via* the mitochondrial-mediated apoptotic pathway.

### Statmp-151 Inhibited Stat3 Phosphorylation

**FIGURE 6:**
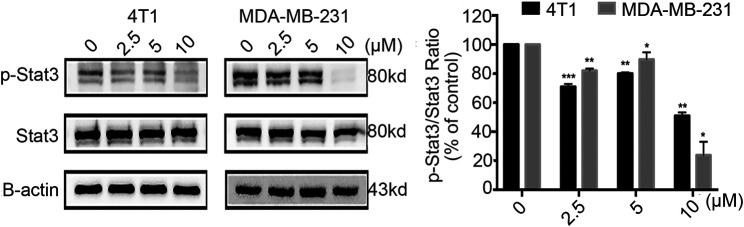
Statmp-151 inhibited phosphorylation of Stat3. The cells treated with Statmp-151 for 24 h were used, and the expression levels of Stat3 and p-Stat3^Tyr705^ were measured by western blotting and the protein expression was quantified.

Phosphorylation by Jak kinase is a key step in the activation of Stat3 (Li et al., 2019); therefore, we detected whether Statmp-151 could influence the expression of p-Stat3. The results confirmed that Statmp-151 could decrease the expression of p-Stat3 but had no effect on total Stat3 in MDA-MB-231 and 4T1 cells Figure 6. The results implied that Statmp-151 inhibited the phosphorylation of Stat3 proteins, consistent with the original drug design.

### Stamp-151 Inhibited the Migration of Breast Cancer Cells

**FIGURE 7:**
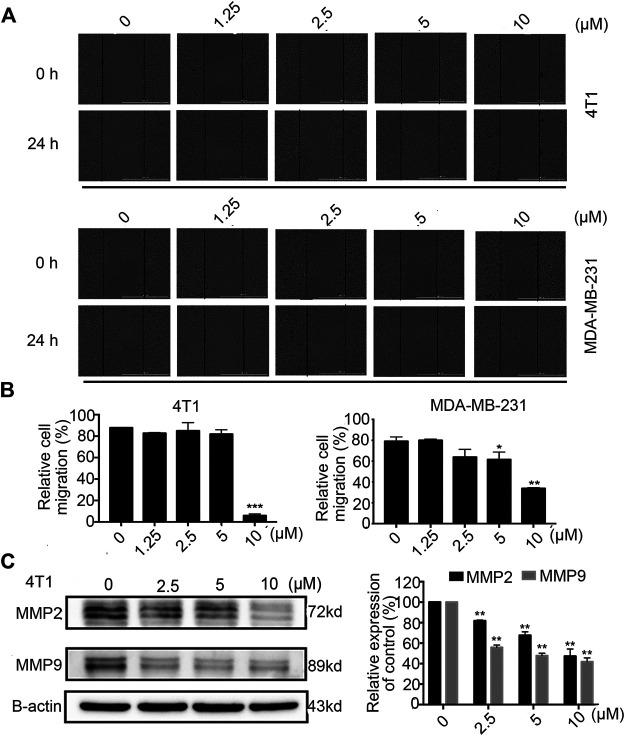
Statmp-151 inhibited cell migration of 4T1 and MDA-MB-231 cells. **(A)** The images were taken after treating with Statmp-151 for 0 and 24 h with the lines indicating the area occupied by the initial scraping. **(B)** The migrated cells were quantified in a histogram. **(C)** The cells treated with Statmp-151 for 24 h were harvested, and the expression levels of MMP-2 and MMP-9 were measured by western blotting and quantified.

Tumour cell migration is one of the committed steps in cancer metastasis (Li et al., 2016). We conducted wound healing assays on 4T1 and MDA-MB-231 cells to explore the therapeutic potential of Statmp-151. As shown in Figure 7A,B, the results indicated that Statmp-151 significantly inhibited the migration of both 4T1 and MDA-MB-231 cells in a concentration-dependent manner. The migration of these cells to the wound area was significantly inhibited after incubation with Statmp-151 for 24 h at 10 µM.

To further verify the mechanism of the antimigratory effects of Statmp-151, we determined the MMP-9 and MMP-2 levels by western blotting. The results suggested that Statmp-151 could significantly inhibit the expression levels of MMP-2 and MMP-9 in 4T1 cells Figure 7C. These results suggested that Statmp-151 possessed a potent ability to inhibit breast cancer cell migration.

### *In Vivo* Anti-breast Cancer Activity of Statmp-151

**FIGURE 8:**
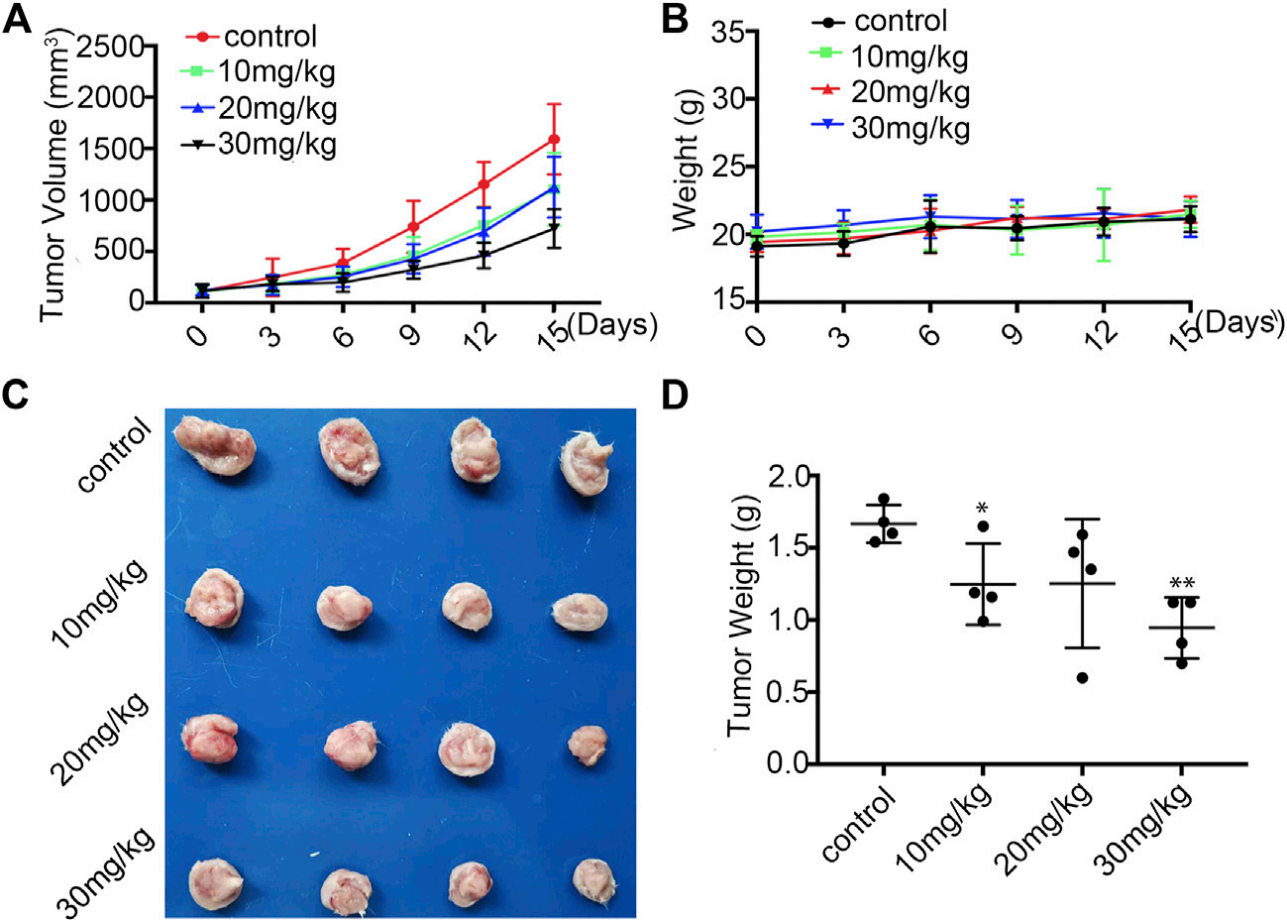
Statmp-151 inhibited tumor growth *in vivo.* **(A)** In 4T1 mice model, the mice were treated with Statmp-151 (10, 20, and 30 mg/kg) or Control. Tumor volumes were measured every 3 days. **(B)** The body weight of 4T1 tumour mice was measured every 3 days. **(C)** Tumours from mice treated with indicated Statmp-151 on the final day (Day 15). **(D)** Tumors weight treated with Statmp-151 at the 15 days.

To evaluate the anti-tumour efficacy of Statmp-151 *in vivo* (Yang et al., 2015), we established a 4T1 tumour-bearing mouse model. The mice were administered Statmp-151 daily at doses of 10, 20 and 30 mg/kg for 15 days (Heppner et al., 2000; Zeng et al., 2020). There was a significant reduction in tumour growth, while the body weight of mice had no significant changes (Figure 8A,B). On the final day, there were significant reductions in tumor size and tumour weight compared with the control group (Figure 8C,D). These results suggested that Statmp-151 had potent anti-tumour activity *in vivo*.

**FIGURE 9:**
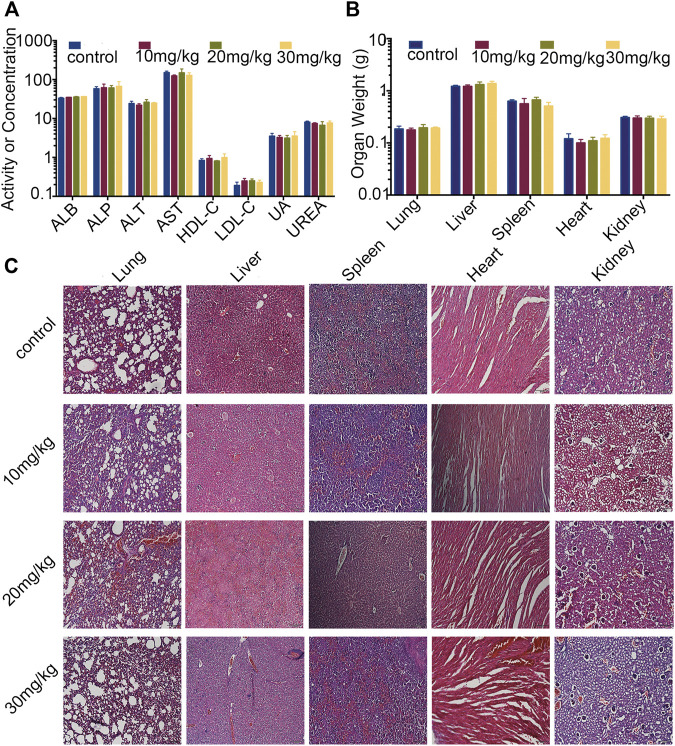
Evaluation of side effects of Statmp-151 in mice. **(A)** Blood biochemical indexes of mice (ALB, ASP unite is g/L, ALT, AST unite is U/L, HDL-C, LDL-C, UREA unite is mmol/l, UA unite is mg/dl). **(B)** The weight of lung, liver, spleen, heart, and kidney. **(C)** Haematoxylin and eosin stained of paraffin sections of lung, liver, spleen, heart and kidney of mice treated with Statmp-151.

As mentioned above, the body weights of the mice were not significantly changed. To further evaluate the safety of Statmp-151, we determined the serum biochemical indexes and weights of the heart, liver, spleen, lung and kidney. Paraffin sections of the lung, liver, spleen, heart and kidney were stained with haematoxylin and eosin. As shown in Figure 9A,B, there were no obvious differences in organ weight or serum biochemical indexes. Furthermore, no pathologic changes were observed in the lung, liver, spleen, heart or kidney compared with those in the control group Figure 9C. Therefore, Statmp-151 was considered to be a relatively safe small molecule compound.

## Discussion

Breast cancer is the most common malignancy in females and is a heterogeneous disease at the molecular level (Harbeck et al., 2019). Accumulating evidence has shown that Stat3 signalling is involved in breast cancer initiation and progression. Inhibition of the activity of Stat3 has become a popular therapeutic strategy (Zhang et al., 2010; Li et al., 2011; Alshaer et al., 2019; Xie et al., 2019). Therefore, the aim of this study was to explore the mechanisms of a novel small molecule inhibitor, Statmp-151, in breast cancer cells and provide support for the treatment of breast cancer.

Stattic is one of the classic inhibitors of Stat3 but is limited by low activity, low selectivity and high toxicity (Zhang et al., 2010). Ligustrazine is an alkaloid found in nature whose structure is simple and easy to modify (Alshaer et al., 2019; Xie et al., 2019). Ligustrazine has good pharmacokinetic properties, fast absorption, broad distribution and no cumulative toxicity (Cheng et al., 2007; Wu et al., 2013; Zeng et al., 2013; Han et al., 2015). In this study, we synthesized a new compound, Statmp-151, by combining ligustrazine and Stattic with the aid of virtual screening and rational drug design.

First, we evaluated the anti-tumour activity of Statmp-151 *in vitro* by MTT assays and colony formation assays. In both tests, Statmp-151 inhibited the proliferation of 4T1, MDA-MB-231, and MCF-7 breast cancer cells in a dose-dependent manner. Then, we verified the apoptosis-inducing effect of Stamp-151 on tumour cells. Our results showed that Stamp-151 induced mitochondrial membrane potential loss and reduced Bcl-2 expression. The steady state of ROS is correlated with the stability of the membrane potential. In view of this, we found that Statmp-151 significantly changed the ROS levels, suggesting that the effect of Statmp-151 on inducing apoptosis was related to changes in ROS levels and mitochondrial membrane potential. Furthermore, we found that Statmp-151 has an anti-tumour effect by inhibiting the phosphorylation of Stat3. Next, we evaluated the effect of Stamp-151 on the migration of breast cancer cells. Wound-healing migration assays confirmed that Statmp-151 could significantly inhibit cell migration. MMP2 and MMP9 are considered to be transport-related proteins, and protein analysis of 4T1 cells showed significant inhibition of MMP2 and MMP9 protein expressions. Our experiments confirmed that Statmp-151 significantly inhibited tumour growth, and there was no significant change in body weight, organ weight, serum biochemistry or haematoxylin-eosin staining in mice, suggesting that Statmp-151 may have no toxic effects *in vivo.*

In summary, Statmp-151 is a small molecule compound that was confirmed to inhibit the growth of breast cancer cells *in vitro* and *in vivo* by blocking Stat3 activation in this study. Based on our preliminary investigations on the various pharmacological properties of Statmp-151, it has good anti-tumour activity and is relatively safe. Additionally, Statmp-151 can undergo continued chemical optimization and pharmacological exploration. Therefore, Statmp-151 is worthy of continued development and is a drug candidate for the treatment of breast cancer.

## Data Availability

The raw data supporting the conclusions of this article will be made available by the authors, without undue reservation.
